# Neural Control of Gas Exchange Patterns in Insects: Locust Density-Dependent Phases as a Test Case

**DOI:** 10.1371/journal.pone.0059967

**Published:** 2013-03-29

**Authors:** Tali S. Berman, Amir Ayali, Eran Gefen

**Affiliations:** 1 Department of Biology and Environment, University of Haifa- Oranim, Tivon, Israel; 2 Department of Zoology, Tel Aviv University, Tel Aviv, Israel; Tokai University, Japan

## Abstract

The adaptive significance of discontinuous gas exchange cycles (DGC) in insects is contentious. Based on observations of DGC occurrence in insects of typically large brain size and often socially-complex life history, and spontaneous DGC in decapitated insects, the neural hypothesis for the evolution of DGC was recently proposed. It posits that DGC is a non-adaptive consequence of adaptive down-regulation of brain activity at rest, reverting ventilatory control to pattern-generating circuits in the thoracic ganglia. In line with the predictions of this new hypothesis, we expected a higher likelihood of DGC in the gregarious phase of the desert locust (*Schistocerca gregaria*, Orthoptera), which is characterized by a larger brain size and increased sensory sensitivity compared with the solitary phase. Furthermore, surgical severing of the neural connections between head and thoracic ganglia was expected to increase DGC prevalence in both phases, and to eliminate phase-dependent variation in gas exchange patterns. Using flow-through respirometry, we measured metabolic rates and gas exchange patterns in locusts at 30°C. In contrast to the predictions of the neural hypothesis, we found no phase-dependent differences in DGC expression. Likewise, surgically severing the descending regulation of thoracic ventilatory control did not increase DGC prevalence in either phase. Moreover, connective-cut solitary locusts abandoned DGC altogether, and employed a typical continuous gas exchange pattern despite maintaining metabolic rate levels of controls. These results are not consistent with the predictions of the neural hypothesis for the evolution of DGC in insects, and instead suggest neural plasticity of ventilatory control.

## Introduction

Insects exchange respiratory gases between their tissues and the external environment through a gas-filled tracheal system. The main tracheae branch off into small-diameter tracheoles, which terminate in close proximity to individual cells. The tracheal system opens to the external environment through paired spiracles, located laterally on the thoracic and abdominal segments of the insect body ]1[. Most insects control spiracular opening through closer/opener muscles, with a resulting variety of gas exchange patterns. These have been categorized as: (i) continuous gas exchange, where spiracles remain constantly opened, or flutter at high-frequency; (ii) cyclic gas exchange, where spiracles open and close intermittently; and (iii) discontinuous gas exchange cycles (DGC), characterized by prolonged closure of the spiracles, during which gas exchange is generally undetectable ]2[.

Despite the extensive research attention it has attracted, DGC have only been found in five insect orders to date: Lepidoptera, Coleoptera, Hymenoptera, Blattodea, and Orthoptera [3], in addition to other tracheated arthropods, such as centipedes, ticks, and solifuges [2]. Moreover, in insects that do exhibit DGC, the pattern was reported to be limited to periods of quiescence (*e.g.* Lepidopteran pupae, or resting adult insects; see [4]). The adaptive value of DGC, contributing to its evolutionary origin and maintenance in insects and other tracheated terrestrial arthropods, is contentious, and has long been the subject of scientific controversy (reviewed by [2], [5]–[7]).

Initially, it was suggested that DGC serves for restricting respiratory water loss rates (“hygric hypothesis”). These lower rates are achieved by a buildup of endotracheal sub-atmospheric pressure during a prolonged period of spiracle closure (C-phase), and a largely inward convective gas transport during a subsequent spiracle-fluttering phase [8]. The “chthonic hypothesis” postulated that DGC has evolved in subterranean insects to promote diffusive respiratory gas exchange in their hypoxic/hypercapnic microhabitats [9]. The “oxidative damage” hypothesis posits that the tracheal system is designed for sufficient respiratory gas transport during activity, and that DGC evolved to maintain sub-normoxic oxygen partial pressure in the tracheal system of resting insects, thus minimizing the risk of tissue oxidative damage [10]. It has also been hypothesized that DGC is not adaptive at all, and results from the interaction of two independent feedback loops responsive to tracheal levels of respiratory gases [11], [12]. In addition, it has been suggested that insect gas exchange patterns form a continuum, where DGCs are limited to periods of low metabolic rates [13].

Most recent is the “neural hypothesis”, suggesting that DGC is a non-adaptive consequence of the energetically adaptive down-regulation of brain activity at rest [4]. It posits that DGC is exhibited in “sleep-like” states (*e.g.*, at rest or during the pupal stage), when central pattern generators in the abdominal and metathoracic ganglia do not receive input from the insect brain [4], [14]. Those authors pointed out that DGC occur in species which typically exhibit large brains (and mushroom bodies in particular), and therefore gain maximal fitness advantage by reducing the energetic costs of brain activity at rest [4]. They also argued that DGC was characteristic of gregarious insects, in which social complexity is correlated with higher likelihood for DGC compared with solitary species. Further supporting evidence for the suggested new adaptive hypothesis included observations of spontaneous DGC following decapitation in ants [15] and cockroaches [14].

The main goal of the present study was to test the neural hypothesis using the desert locust *Schistocerca gregaria* (Orthoptera: Acrididae) as a model. Locust density-dependent phase-polyphenism allows testing predictions of the neural hypothesis without the need for rigorous phylogenetic information required for comparative studies [16]. Well studied differences between the gregarious and solitary phases of *S. gregaria* [17] include the active aggregation behavior of the former, as well as a multitude of neurological differences (*e.g.* [18], reviewed in [17]), including 30% larger brain size in gregarious compared to solitary locusts despite their similar body size [19].

We therefore monitored gas exchange patterns in gregarious and solitary *S. gregaria*, expecting higher DGC prevalence at rest in the former, based on both brain size and level of sociality. We then severed the neural pathways connecting head and thoracic ganglia, in order to monitor the effect on respiratory gas exchange patterns, and test the prediction that removal of inhibitory brain output would result in a shift towards increased DGC prevalence. This work is, to our knowledge, the first to experimentally test the predictions of the neural hypothesis for the evolution of DGC in insects. Our findings are not consistent with these predictions, but instead suggest neural plasticity of ventilatory control.

## Materials and Methods

Gregarious *S. gregaria* originated from stock populations at the Tel Aviv University, where 1-generation solitary rearing has been shown to elicit significant phase-dependent differences in sensory and central neural elements [18]. At the University of Haifa-Oranim, gregarious locusts were held in stock cages. Hatchlings of the same egg clutches were randomly distributed to either crowded conditions or, alternatively, in order to obtain solitary locusts, placed in individual chambers within 3 hr of hatching. The locusts were fed fresh grass and dry oats *ad lib*, and kept at 33.0±3.0°C and 12L:12D photoperiod in separate rooms until adult emergence.

**Figure 1:**
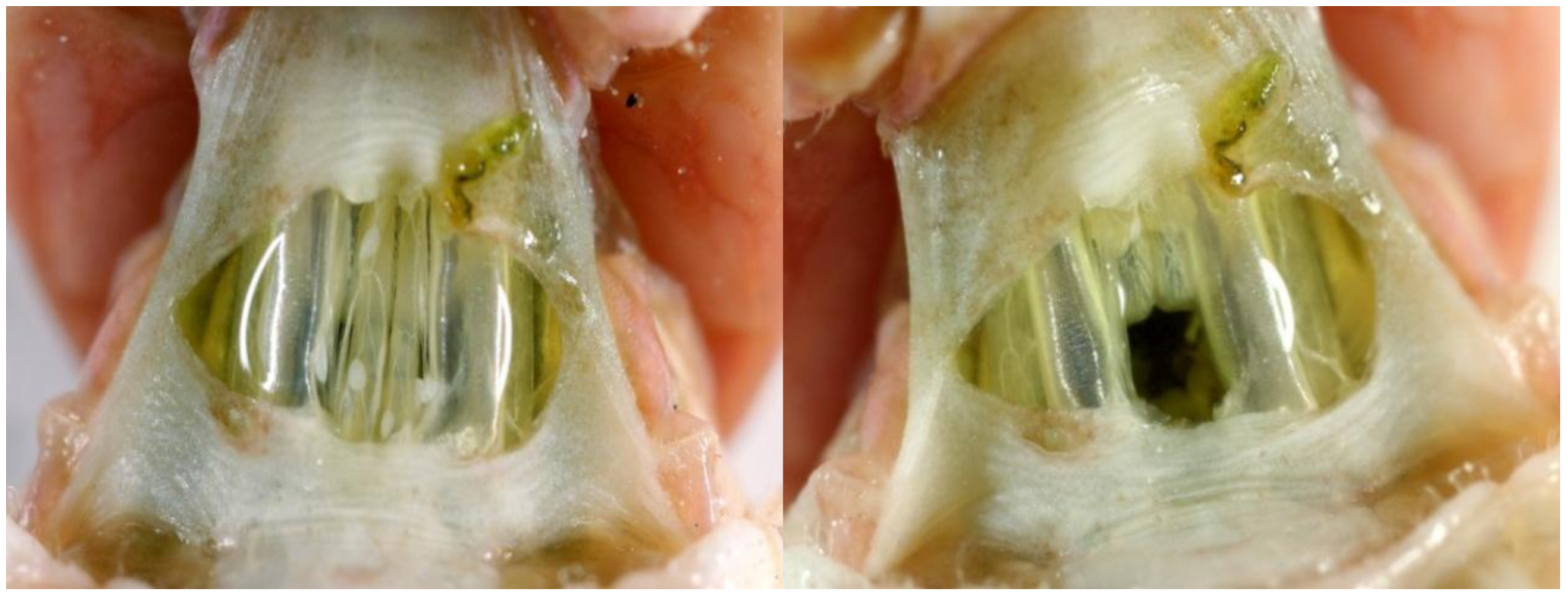
Connectives between head and thoracic ganglia before (left) and after (right) lesion. Ventral view of the locust neck incision exposing the connectives between head and thoracic ganglia before (left) and after (right) lesion. Following surgery the locusts were allowed 14–24 h for recovery, during which access to food was denied, prior to respirometry.

Adult solitary and gregarious locusts were transferred to separate incubators, (Friocell 222, MMM, Munich, Germany, and DT2-MP38, Tritech Research, Los Angeles, CA, USA, respectively) where they were kept at 30.0±0.2°C and 14L:10D photoperiod and fed *ad lib* until experimentation. The experimental design comprised three treatments for each developmental density-dependent phase: intact; connective-cut (CC); and sham-operated (SO). Cross-incision of the ventral neck membrane enabled cutting of the connectives, thus disconnecting the head and thoracic ganglia in the CC group (Fig. 1). A similar procedure, without nervous lesion, was carried out in the SO group. In order to prevent bleeding in locusts of both treatments, the genae were fixed to the thorax with wax. Surgical procedures were carried out one day prior to respirometry at ∼17∶00, at which time locusts of all three treatments were prevented further access to food. Only male locusts were used in the study.

Flow-through respirometry was used for measurement of CO_2_ emission rates (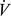
CO_2_) and determination of gas exchange patterns. The locusts were introduced into darkened glass metabolic chambers (1.8 cm diameter×9 cm length, volume ∼23 mL), placed horizontally in the incubator at 30.0±0.2°C. Air was passed through silica gel/Ascarite® to the chamber at 100 mL·min^−1^, using mass flow controllers (FMA 2617A; Omega Engineering, Stamford, CT, USA). The locusts were left undisturbed for ∼90 min before the excurrent air was passed through a pre-calibrated LI-7000 CO_2_/H_2_O analyzer (LI-COR Bioscience, Lincoln, NE, USA). Data were recorded for an additional 90 min and analyzed using Expedata acquisition and analysis software (Sable Systems International, Las Vegas, NV, USA). Baselines were recorded at the beginning and end of every individual recording, by passing dry CO_2_-free air directly through the analyzer. Locusts were monitored for 48 h after measurements, and results for the two individuals (out of a total of 135) that died during that period were discarded.

In order to avoid biased categorization of gas exchange patterns, a threshold 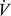
CO_2_ value was determined, under which the spiracles were assumed to be in a C-phase. A separate sample of 9 individuals was used for measuring 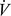
CO_2_ in 100% oxygen, conditions promoting extended uninterrupted spiracle closure; and mean emission rates during these periods were determined (54.7±4.4 µL·g^−1^·h^−1^). These values were doubled to provide a threshold value for determination of C-phase in normoxia, in order to account for instrumental noise [20]. Gas exchange pattern was categorized as DGC whenever at least 4 successive cycles included a C-phase lasting at least 90 sec. Cyclic and continuous exchanges were categorized by inspection.

Statistical analysis was performed using SPSS 19.0 statistical software (IBM). Exact tests were performed when expected values were too small for χ^2^ tests [21]. Values given throughout the report are means ± s.e.m unless stated otherwise.

## Results

Preliminary measurements at 25°C did not indicate phase-dependent differences in gas exchange patterns, with more than 80% of both solitary and gregarious locusts exhibiting DGC (data not shown). Therefore, we continued our experiments at 30°C, hypothesizing that increased temperature would further enhance the disproportionately high energetic cost of brain activity [19], and thus trigger increased DGC expression in gregarious locusts. At this experimental temperature, 69 gregarious and 64 solitary individuals were used (body mass of 1.316±0.017 g and 1.418±0.030 g, respectively). In contrast to our prediction for intact individuals, higher DGC prevalence was recorded among the solitary group (Table 1), although phase-dependent variation in gas exchange patterns among intact specimens was not statistically significant (χ^2^_(df = 2)_ = 2.647, P = 0.266).

**Table 1:** Gas exchange pattern distribution across the two locust phases and three experimental treatments (N = 133).

phase	treatment	Gas exchange pattern		
		Continuous	Cyclic	DGC
gregarious	intact	9	7	9
	sham-operated (SO)	5	5	12
	connective-cut (CC)	9	7	6
solitary	intact	4	4	12
	sham-operated (SO)	7	5	10
	connective-cut (CC)	21	1	0

No significant differences were found between SO and intact individuals in either density-dependent phase (P>0.60). Cutting the connectives had a significant effect on gas exchange pattern in solitary (P<0.001) but not gregarious (P = 0.38) locusts.

**Figure 2:**
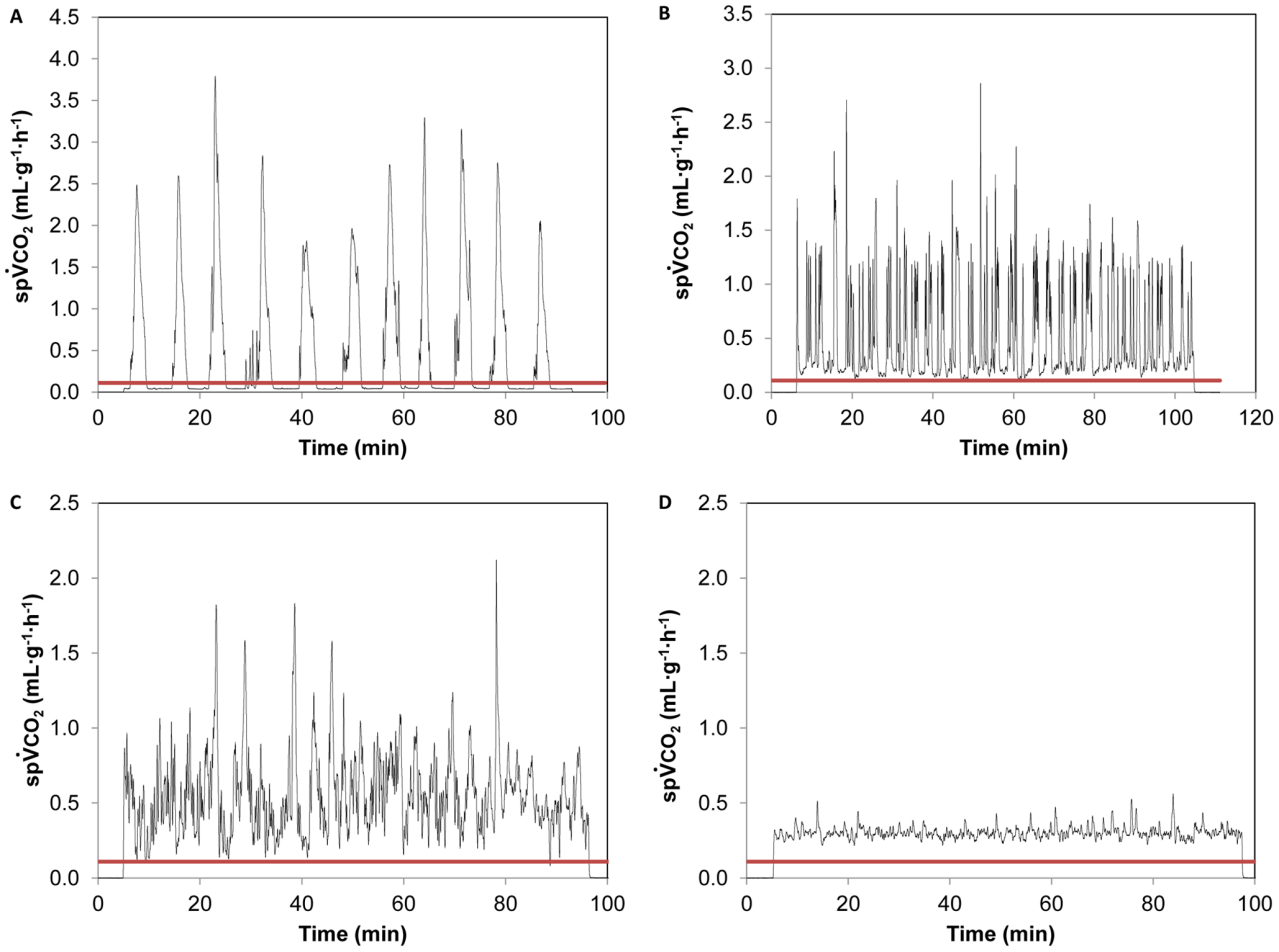
Gas exchange patterns in connective-cut *S. gregaria* of both density-dependent phases, measured at 30°C. Connective-cut gregarious locusts exhibited all three patterns (a, b, c: DGC, cyclic and continuous, respectively), at similar proportions compared with controls. Connective-cut solitary locusts abandoned DGC and displayed a typical continuous gas exchange pattern (d). Red lines indicate threshold value for determination of spiracle closure (see methods).

Sham-operated locusts of the two phases exhibited similar gas exchange pattern ratios (χ^2^_(df = 2)_ = 0.515, P = 0.773), which did not differ from those of intact individuals within treatment (χ^2^_(df = 2)_ = 1.720, P = 0.423 and P = 0.607 for gregarious and solitary, respectively). We therefore pooled both control treatments for greater statistical power when comparing gas exchange pattern distribution to that of connective-cut (CC) individuals. The CC gregarious locusts still did not differ significantly from controls in gas exchange pattern distribution (χ^2^_(df = 2)_ = 1.932, P = 0.381), with >25% expressing DGC (See traces in Fig. 2A–C). In contrast, CC solitary locusts abolished DGC altogether, and thus differed significantly in pattern distribution from controls (χ^2^_(df = 2)_ = 28.010, P<0.001) and CC gregaria (exact test, P<0.001). Interestingly, calculated 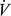
CO_2_ coefficient of variation during the last 60 minutes of continuous gas exchange was 24.6±2.5% (N = 21) for CC solitary locusts (see Fig. 2D), compared with a range of 44.2±7.6% to 47.5±3.9% (*e.g.* Fig. 2C) for all other five phase×treatment combinations (see Table 1 for sample sizes).

No significant phase-dependent differences were found in 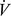
CO_2_ of intact locusts (ANCOVA, with body mass as a covariate; F_1,42_ = 1.554, P = 0.220). Metabolic rates of intact gregarious locusts expressing DGC (454.2±29.5 µL·g^−1^·h^−1^; see Table 1 for sample sizes) were lower in comparison with individuals exchanging respiratory gases continuously (610.2±61.1 µL·g^−1^·h^−1^), with cyclic exchange characterized by intermediate levels (471.8±50.2 µL·g^−1^·h^−1^), but these differences were just short of statistical significance (F_2,21_ = 3.458, P = 0.050). A similar pattern was observed in intact solitary locusts (487.8±21.5 µL·g^−1^·h^−1^ and 409.5±18.0 µL·g^−1^·h^−1^ for continuous exchange and DGC, respectively; F_2,16_ = 2.239, P = 0.139).

**Figure 3:**
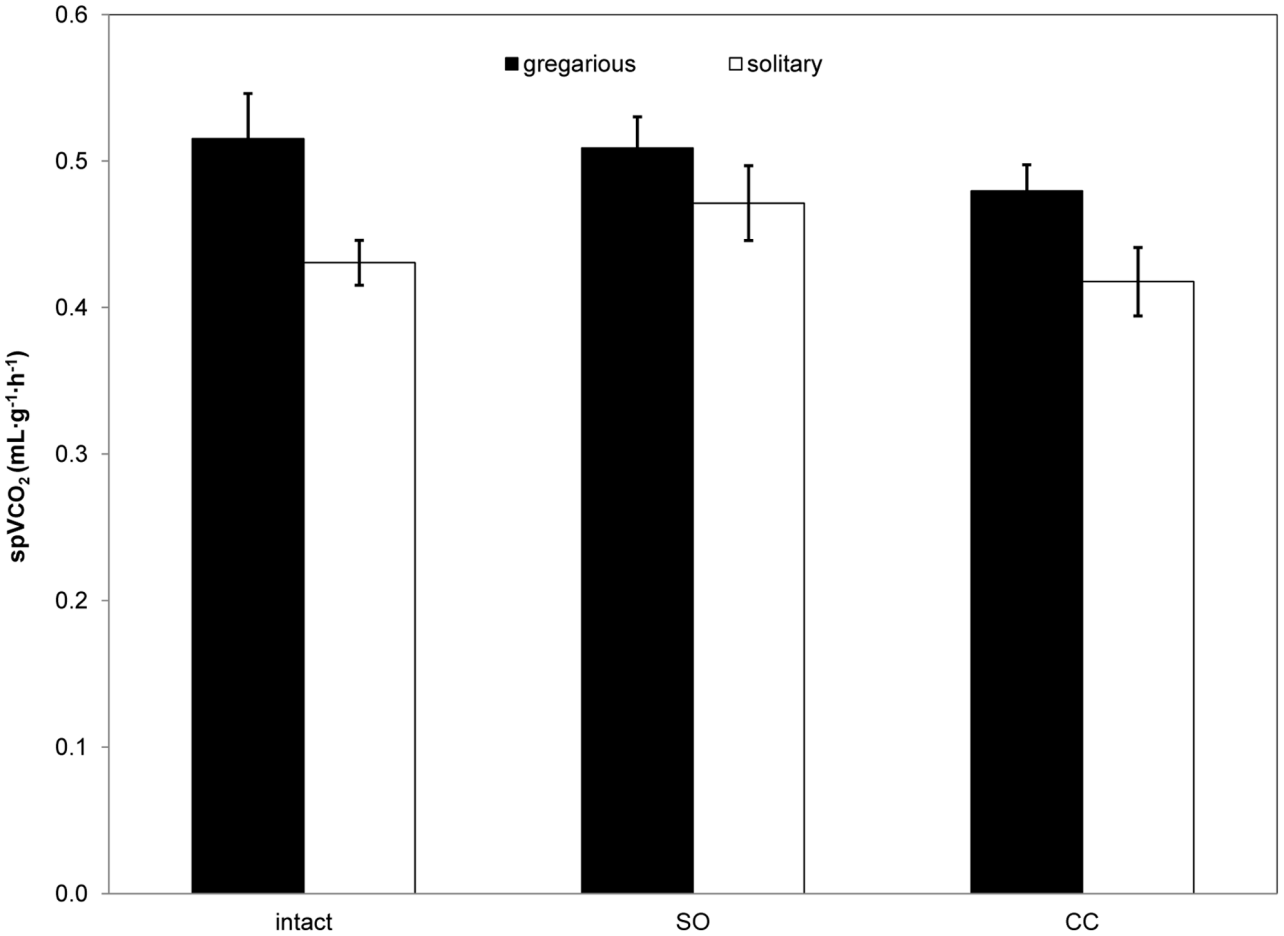
Mass-specific metabolic rates of gregarious and solitary locusts across experimental treatments. Mean (±s.e.m.) mass-specific CO_2_ emission rates (sp
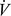
CO_2_) of intact, sham-operated (SO), and connective-cut (CC) *S. gregaria* of both phases, measured at 30°C. Similar rates were measured for intact gregarious and solitary intact locusts (ANCOVA with body mass as a covariate, P = 0.22), but significant phase effect was found across treatments (Two-way ANCOVA, P = 0.003).

Comparison of 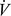
CO_2_ across treatments indicated significantly higher rates for gregarious compared with solitary locusts (F_1,126_ = 8.975, P = 0.003; Fig. 3), whereas treatment effect (F_2,126_ = 1.445, P = 0.240) and phase×treatment interaction (F_2,126_ = 0.125, P = 0.883) were not significant.

## Discussion

Locusts and grasshoppers offer an excellent example of what has proved to be an elusive unequivocal adaptive explanation for the evolution and maintenance of DGC in insects. The classic hygric hypothesis is at odds with the observation that the grasshopper *Romalea guttata* abandoned DGC when dehydrated [22], a condition which is expected to favor water conservation. Resting *Schistocerca americana* were shown to regulate tracheal oxygen partial pressure at near normoxic levels [23], which does not lend support to the oxidative damage hypothesis either. The superterranean existence of grasshoppers and locusts make the chthonic hypothesis an unlikely explanation for the adaptive value of DGC in these insects. Therefore, locusts provide an attractive model for testing new alternative hypotheses.

To the best of our knowledge, this is the first study to test predictions of the recently proposed neural hypothesis [4]. Among the circumstantial evidence lending support to the neural hypothesis, are the typically larger brain sizes in species belonging to the five insect orders in which DGC has been observed. In addition, interspecific comparison among cockroaches suggested that DGC correlates with gregarious behavior [4]. In this study we used phase-polyphenism in locusts in order to compare gregarious and solitary individuals, while avoiding potentially confounding phylogenetic effects inherent to interspecific comparisons [16]. Moreover, gregarious *S. gregaria* have a larger brain size [19] and higher neural sensitivity (reviewed in [17]) compared with solitary locusts, both of which favor DGC at rest according to the neural hypothesis. However, we did not find phase-dependent differences in the distribution of gas exchange patterns in either intact or SO samples (Table 1) at 25 or 30°C. Although we did not find a difference in 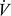
CO_2_ between intact gregarious and solitary locusts, a highly significant phase effect on 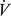
CO_2_ over the entire dataset (p<0.001) may suggest that higher metabolic rates in gregarious locusts confound the phase effect on the gas exchange pattern. According to the neural hypothesis, DGC is expected to be more prevalent among gregarious forms [4], which was not reflected in our results. At the same time, the higher metabolic rates of gregarious in comparison with solitary locusts at rest are expected to decrease the likelihood for DGC in the former [20], as reflected in within-phase correlated gas exchange and metabolic rate patterns in intact individuals.

Additional supporting evidence for the neural hypothesis is provided by the spontaneous expression of DGC in decapitated ants [15] and cockroaches [14]. In the present study, by severing the connectives between head and thoracic ganglia, we released the thoracic ganglia from descending control of ventilatory activity, and thus directly tested a fundamental component of the neural hypothesis: namely, the effect of neural connection between the brain and thoracic ganglia on gas exchange patterns. In contrast with the predictions, we did not observe a higher prevalence of DGC in CC *S. gregaria*. The distribution of gas exchange patterns among gregarious locusts was similar to that observed among controls, whereas solitary locusts abolished DGC altogether (Table 1). Indeed, the latter appeared to abandon any intermittent gas exchange pattern, and exhibited a profound continuous pattern characterized by highly stable 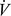
CO_2_ (Fig. 2D). Importantly, there were no significant treatment or phase×treatment effects on metabolic rates which could explain the variation in gas exchange pattern distribution among CC gregarious and solitary locusts. Evidently, the almost exclusive (21 of 22 individuals; Table 1) use of continuous gas exchange among CC solitary locusts was not a result of increased metabolic rates (Fig. 3). In addition, a similar gas exchange pattern distribution among SO and intact locusts in both phases confirmed that neck incision had no effect on gas exchange patterns, and the observed changes in ventilatory pattern were caused by severing the neural connections in solitary locusts.

According to the neural hypothesis DGC results from the “release” of central pattern generating neural circuits in thoracic ganglia from descending inhibition originating from head ganglia. However, results in this study indicate that DGC is not inhibited by input from the brain in resting *S. gregaria*. Furthermore, the effect of head ganglia input on thoracic ventilatory pattern generators is phase-specific, as DGC expression in solitary locusts depends on intact neural circuitry. Our findings indicate that the neural hypothesis, like other previously-proposed adaptive hypotheses, cannot unequivocally account for the evolution of DGC in insects. The intriguing difference in the effect of severing the head ganglia on gas exchange control in hymenoptera and Blattodea on the one hand, and the orthopteran *S. gregaria* on the other hand, is in accordance with the finding that DGC has evolved independently in insects at least five times [3]. Nevertheless, our findings highlight the plasticity of neural control of ventilation in locusts, which could potentially provide valuable insights into the evolution of DGC in insects.
